# Translating blood-based biomarkers into Alzheimer’s disease clinical practice: screening, diagnosis, and longitudinal monitoring

**DOI:** 10.3389/fnagi.2026.1852137

**Published:** 2026-08-21

**Authors:** Binbin Xie, Wang Zhang, Yu Zhang, Li Liu, Junjie You, Qiong Li

**Affiliations:** 1 Department of Neurology, Chongqing Mental Health Center, Chongqing, China; 2 Department of Geriatrics, Chongqing Mental Health Center, Chongqing, China; 3 Department of Hospital-Acquired Infection Control, Chongqing Mental Health Center, Chongqing, China

**Keywords:** Alzheimer’s disease, amyloid-β, blood-based biomarkers, clinical implementation, glial fibrillary acidic protein, neurofilament light chain, phosphorylated tau

## Abstract

Blood-based biomarkers (BBMs) are increasingly being integrated into Alzheimer’s disease (AD) clinical pathways by providing scalable tools to track amyloid, tau, neurodegeneration, and inflammation dimensions of the ATN-I framework. As biological characterization and biomarker-informed assessment are increasingly integrated into AD care, there is growing interest in clinically applicable blood-based approaches capable of reflecting AD-related biological changes across different disease stages. This review examines recent advances in BBMs through the ATN-I framework while emphasizing their clinical decision value, analytical feasibility, economic considerations, and implementation barriers across different healthcare settings. Building on these findings, we examine the evidence supporting clinical use, with a focus on appropriate use criteria, clinical validation, laboratory feasibility, and consistent interpretation methods. Furthermore, this review explores how integrating BBMs with traditional cerebrospinal fluid and neuroimaging tests may support symptom-driven clinical triage, probability refinement, and early evaluation in selected individuals, improving clinical workflow efficiency and accessibility. Finally, the article addresses remaining challenges related to clinical validation, platform accessibility, cost-effectiveness, and equitable deployment, highlighting how emerging biomarker strategies may contribute to more scalable AD assessment.

## 1 Introduction

Alzheimer’s disease (AD), the leading cause of dementia, represents a substantial and growing global health burden, with prevalence projected to exceed 150 million cases by 2050 (Scheltens et al., 2021; GBD 2019 Dementia Forecasting Collaborators, 2022; Twarowski and Herbet, 2023). Despite advances in therapeutic development, disease-modifying treatments remain challenging to implement effectively, partly because pathological changes begin years before clinical manifestations become apparent (Kapogiannis, 2020; Zuliani et al., 2026). This prolonged interval between biological alteration and symptom expression highlights the need for accessible approaches that enable earlier biological characterization and risk assessment.

Beyond amyloid-β (Aβ) and tau, biomarkers capturing downstream axonal injury, synaptic dysfunction, and glial activation are increasingly utilized for biological staging, prognostic assessment, and treatment monitoring (Blennow et al., 1995; Jack et al., 2024; Mielke et al., 2024b). Within this framework, biomarkers have become important indicators of *in vivo* pathology that complement clinical assessment and other diagnostic modalities, evolving from supportive tools to key components of biological characterization (Biomarkers Definitions Working Group, 2001; Jack et al., 2018). Neuroimaging modalities, including magnetic resonance imaging and positron emission tomography (PET), and cerebrospinal fluid (CSF) biomarkers provide robust measures of neurodegeneration and amyloid/tau pathology, but remain constrained by cost, invasiveness, and limited scalability for serial or high-volume assessment within clinically selected populations (Olsson et al., 2016; Koenig et al., 2018; Hansson et al., 2019; Ossenkoppele et al., 2022). This has revealed a critical gap between biologically accurate disease definition and clinically deployable diagnostic systems.

Longitudinal and neuropathological studies demonstrate that Aβ and tau accumulation begin more than a decade before clinical symptom onset, defining a prolonged preclinical phase of silent disease progression (Reijner et al., 2025; Shi et al., 2025). Large cohort studies further confirm that these molecular changes evolve continuously over time, providing a wide but clinically inaccessible therapeutic window (Jia et al., 2024; Li et al., 2024). However, the inability of CSF and PET to provide scalable, minimally invasive, and repeatable assessment across this continuum represents a major translational bottleneck. This limitation has driven increasing interest in blood-based biomarkers (BBMs), which enable peripheral sampling of central pathological processes and are emerging as scalable tools for early biological characterization, risk assessment, and longitudinal monitoring within appropriate clinical contexts (Leuzy et al., 2022; Jack et al., 2024). Despite rapid advances, research on BBMs in AD remains fragmented across classes, platforms, and applications. Critically, their clinical integration depends on a coherent synthesis spanning pathological axes and economic viability. These translational imperatives are further complicated by real-world healthcare heterogeneity. Across healthcare systems, BBM implementation is shaped by population diversity, diagnostic capacity, and healthcare infrastructure (Mielke et al., 2024a; Schöll et al., 2024; Rodda et al., 2025). China exemplifies these challenges, with emerging but still relatively limited population-specific evidence compared with Western cohorts, highlighting the need for additional validation studies, reference intervals, and clinical integration of BBMs in Chinese populations (Gao et al., 2023; Jiang et al., 2025).

This clinically oriented review provides a narrative overview of current evidence across amyloid, tau, neurodegeneration, and neuroinflammation pathways, evaluating their respective roles in symptom-driven triage and early evaluation, diagnosis, and monitoring. In addition to discussing the existing literature on BBMs, this review considers clinical applicability, probabilistic interpretation, implementation considerations, and platform-related economic factors within a translational framework for AD. The review further discusses translational readiness, implementation barriers, and future directions.

## 2 The ATN-I framework in blood: biological rationale and analytical landscape, and translational considerations

The amyloid, tau, neurodegeneration, and inflammation/glial activation framework (ATN-I) provides a biological basis for interpreting BBMs across distinct pathological domains, including amyloid pathology (A), tau pathology (T), neurodegeneration (N), and inflammation/glial activation (I) in AD (Table 1; Jack et al., 2018; Imbimbo et al., 2023; Jack et al., 2024). However, translation into clinical practice requires consideration of analytical performance, clinical applicability, and implementation feasibility beyond biological classification.

**TABLE 1:** Clinical application framework of ATN-I BBMs in AD.

Clinical setting	Clinical purpose	Target population	Biomarker axis	BBMs	Clinical scenario	Interpretation principle	Major confounders	Key limitations
Primary care risk assessment or triage	Risk refinement or rule-out	Post-assessment older adults with cognitive concerns	A (±I)	Aβ42/40, GFAP	Initial cognitive concern	Probability modifier (not diagnostic)	Age, inflammation, CKD	Low specificity; not standalone
Symptom-triggered community triage	Pre-referral stratification	Older adults with cognitive concerns / clinically defined risk	A + I	Aβ42/40, GFAP	Post-assessment risk refinement	Modifies AD-pathology probability and informs referral	Vascular disease, systemic inflammation	High false-positive risk
Memory clinic triage	AD likelihood stratification	MCI/early cognitive impairment	A + T	p-tau217, Aβ42/40	PET/CSF referral triage	Two-threshold probabilistic model	Ethnicity, assay platform variability	Cutoff instability across labs
Specialist diagnostic workup	Diagnostic support (adjunctive)	Symptomatic patients with unclear etiology	A + T + I	p-tau217, p-tau181, GFAP, Aβ42/40	Differential diagnosis in dementia syndromes	Multimodal probabilistic integration	Mixed pathology, CKD, cerebrovascular disease	Cannot replace confirmatory testing
Neurology follow-up	Disease progression monitoring	Diagnosed AD / MCI	N	NfL	Longitudinal monitoring in clinic	Longitudinal change prioritized	Stroke, trauma, renal disease	Non-specific to AD
Neuroinflammation monitoring	Adjunct biological monitoring	Preclinical / symptomatic AD	I	GFAP	Research/selected follow-up	Astrocytic activation proxy	Age, blood–brain barrier disruption	Limited disease specificity
Multimodal ATN-I profiling	Biological characterization	Research / specialty cohorts	A + T + N + I	Full panel	Precision medicine stratification	Systems-level, non-stage interpretation	All systemic comorbidities	Requires harmonized assays
Clinical trial enrichment	Enrollment selection	Preclinical / prodromal AD	A + T	p-tau217, Aβ42/40	Trial enrichment or eligibility	Probability-based inclusion	Population heterogeneity	Trial-specific thresholds
Treatment monitoring	Pharmacodynamic response	Trial / treated AD patients	A + T + N + I	p-tau217, GFAP, NfL	Drug response monitoring	Group-level biological change	Intercurrent illness, assay drift	Not validated surrogate endpoint

This table presents a narrative, context-dependent framework for the clinical application of ATN-I BBMs in AD and is not intended as a standardized clinical workflow or diagnostic algorithm. ATN-I represents biological processes rather than disease stages. CSF and PET remain reference standards in uncertain or discordant cases. AD, Alzheimer’s disease; BBMs, blood-based biomarkers; Aβ, amyloid-β; CSF, cerebrospinal fluid; PET, positron emission tomography; A, amyloid; T, tau; N, neurodegeneration; I, inflammation; p-tau, phosphorylated tau; CKD, chronic kidney disease; MCI, mild cognitive impairment; NfL, neurofilament light chain; GFAP, glial fibrillary acidic protein.

### 2.1 Aβ axis: predicting cerebral amyloidosis

Plasma Aβ is primarily considered an amyloid-enrichment biomarker rather than a standalone diagnostic marker. Although its association with cerebral amyloid pathology is supported by isotope-labeling and imaging studies (Ovod et al., 2017), its clinical interpretation remains dependent on assay performance, population characteristics, and integration with other biomarkers. Because absolute plasma Aβ42 concentrations demonstrate substantial biological and analytical variability, the Aβ42/40 ratio has become a commonly used approach, providing improved robustness across individuals and analytical platforms (Cai et al., 2023). Plasma Aβ42/40 therefore has potential value for initial amyloid risk estimation and patient stratification rather than direct confirmation of AD pathology (Hansson et al., 2019). Clinical studies indicate that plasma Aβ42/40 provides moderate discrimination of amyloid-related pathology and may support enrichment strategies in clinically selected populations (Chen et al., 2021). Postmortem and imaging studies further suggest that plasma Aβ42/40 is associated with amyloid plaque burden, although the strength of this association varies across cohorts and analytical platforms (Brickman et al., 2021; Salvadó et al., 2023). From a translational perspective, the major clinical value of plasma Aβ42/40 lies in probability refinement and triage among clinically selected individuals, helping prioritize the use of reference-standard procedures rather than replacing established amyloid assessment methods. In research settings, plasma Aβ42/40 has also been proposed as a trial-enrichment biomarker for recruiting participants into preclinical and prodromal AD studies (Schindler et al., 2019), and its longitudinal trajectory may also help evaluate therapeutic effects in precision-treatment settings (McDade et al., 2022; Sims et al., 2023).

Several analytical platforms are currently available for plasma Aβ measurement (Table 2), including conventional immunoassays such as enzyme-linked immunosorbent assays (ELISA) and Luminex xMAP-based assays, ultrasensitive platforms such as single-molecule array (Simoa) digital immunoassays, fully automated electrochemiluminescence immunoassays (ECLIA; e.g., Roche Elecsys), immunomagnetic reduction (IMR), and mass spectrometry (MS)-based approaches (De Meyer et al., 2020; Janelidze et al., 2021; Leuzy et al., 2022). These platforms differ in analytical sensitivity, specificity, scalability, and clinical feasibility, and their selection depends largely on the intended application rather than the superiority of a single technology. Head-to-head comparisons of plasma Aβ42/40 assays have demonstrated substantial inter-platform variability, with MS-based methods generally showing higher analytical specificity, whereas automated immunoassays such as Roche Elecsys provide advantages in scalability, throughput, and integration into routine laboratory workflows (Janelidze et al., 2021; Keshavan et al., 2021; Udeh-Momoh et al., 2022; Pascual-Lucas et al., 2023). ELISA has been widely used in early plasma Aβ research because of its accessibility and low cost, but its lower sensitivity and higher variability have restricted clinical use; it now serves mainly as a research tool for comparing newer platforms (De Meyer et al., 2020; Leuzy et al., 2022). Luminex-based assays also contributed to early plasma Aβ research, although their clinical translation has been limited by sensitivity and preanalytical variability (Toledo et al., 2011; Chen et al., 2023). Ultrasensitive platforms, particularly Simoa and MS, have improved detection of low-abundance Aβ species and demonstrated stronger associations with amyloid PET status (Lue et al., 2017; Chatterjee et al., 2019). Simoa-based plasma Aβ42/40 measurement showed high negative predictive value for cerebral amyloidosis and may reduce PET requirements during clinical trial recruitment (De Meyer et al., 2020). Fully automated platforms offer advantages in scalability and clinical laboratory integration. For example, plasma Aβ42/40 assays based on chemiluminescent enzyme immunoassay (CLEIA) technology have shown high performance in identifying Aβ PET-positive individuals (Bun et al., 2023). Successful clinical implementation will therefore depend on selecting platforms that balance analytical performance and operational feasibility.

**TABLE 2:** Two-layer translational framework of BBM analytical platforms in AD.

Biomarker target(s)	Analytical platform	Analytical strength	Translational role	Current use setting	Limitations and barriers	Economic considerations
Layer 1. Clinically deployable or translationally advanced platforms						
Aβ42/40 ratio	Simoa	Ultrasensitive low-abundance detection	Research enrichment and longitudinal risk profiling	Memory clinics / research cohorts	Preanalytical variability; dedicated instrumentation; local validation needed	High reagent/instrument costs limit scale-up
	Automated ECLIA (e.g., Elecsys)	Fully automated, reproducible, and scalable	Selected triage and clinical translation	Clinical laboratories / memory clinics	Platform-specific calibration; cross-site harmonization needed	Existing infrastructure enables cost-efficient scaling
	CLEIA platform (e.g., HISCL)	Automated, sensitive, high-throughput	Preliminary amyloid-likelihood triage	Memory clinics / hospital labs	Population-specific cutoffs; local validation	Automation improves operational efficiency
	LC-MS/MS	High specificity, low matrix interference	Reference analysis, validation, and stratification	Specialized centers / research hospitals	Low throughput; complex workflow; specialized infrastructure	High capital/staffing costs limit scale-up
p-tau217, p-tau181	Simoa	High sensitivity; broad dynamic range	Probability stratification and rule-in support	Memory clinics / specialist centers	Assay-specific thresholds; workflow validation needed	Higher costs than automated analyzers
	ECL immunoassay (MSD)	Cross-cohort reproducibility	Diagnostic clarification support	Clinical laboratories	Cross-platform harmonization remains challenging	Automation supports clinical scaling
	Lumipulse CLEIA platform	Automated and clinically compatible	Triage and diagnostic clarification	Hospital laboratories / specialist centers	Clinical validation; standardized reporting needed	Compatible with routine laboratories
	CLEIA platform (e.g., HISCL)	Automated, sensitive p-tau detection	Clinical triage and biological characterization	Memory clinics / specialty centers	Calibration variability; population effects; standardization needed	Automation supports cost-efficient implementation
NfL	Simoa	Ultrasensitive axonal-injury detection	Progression and longitudinal monitoring	Neurology follow-up clinics	Limited AD specificity; contextual interpretation required	Platform costs may limit routine use
	Lumipulse G NfL Blood (automated CLEIA)	Fully automated, high-throughput	Longitudinal monitoring	Clinical laboratories / neurology settings	Limited AD specificity; biological confounders	Supports routine scale-up
	Ella (microfluidic immunoassay)	Scalable and clinically usable	Longitudinal monitoring	Clinical laboratories	Cross-platform standardization remains incomplete	Cartridge workflow may support scale-up
GFAP	Simoa	Sensitive astrocytic-activation detection	Preclinical characterization and stratification	Memory clinics / research cohorts	Low specificity; multimodal interpretation required	Ultrasensitive testing increases costs
	Lumipulse G GFAP (automated CLEIA; RUO)	Fully automated, high-throughput	Translational assessment	Research / translational settings	RUO; broader validation needed	Potential for future scale-up
	Ella (microfluidic immunoassay)	High-throughput clinical scalability	Clinical translation and routine stratification	Clinical laboratories	Standardization; broader validation needed	Automation may improve clinical efficiency
Layer 2. Research-stage, exploratory, or emerging platforms						
Aβ42/40 ratio	ELISA	Low-cost and accessible	Exploratory low-resource assessment	Research / limited-resource settings	Lower sensitivity/reproducibility limit adoption	Low setup costs suit limited-resource settings
	Luminex xMAP	Low-volume multiplexing	Exploratory multiplex profiling	Research settings	Matrix effects; limited sensitivity restrict translation	Efficient multiplexing; limited routine scalability
p-tau217, p-tau181, and eMTBR-tau243	LC-MS/MS	Specific multiprotein/multisite analysis	Mechanistic staging and advanced profiling	Translational research centers	Complex workflow; limited access	Infrastructure/labor demands limit scalability
	Simoa (mid-region-targeted for p-tau181)	Potentially greater tau specificity	Exploratory tau-biomarker refinement	Research settings	Limited multicenter validation	High platform costs may limit routine use
	IMR	Sensitive low-abundance tau detection	Exploratory tau detection; proof-of-concept studies	Research laboratories / regional centers	Limited validation/standardization; multicenter reproducibility uncertain	Specialized instrumentation; scalability uncertain
	IP-MS (eMTBR-tau243)	High specificity and precision	Tau-pathology characterization and anti-tau trial selection	Research settings	Complex preparation; specialized laboratories required	High capital costs limit implementation
	NULISA	Sensitive multiplexing	Panel discovery and phenotyping	Research settings	Limited clinical standardization and interpretation frameworks	Economic scalability remains unproven
	SMID	Simoa-comparable sensitivity; multiplexing	Chinese-population clinical translation	Memory clinics / hospital labs	Limited population validation and longitudinal evidence	Potential local access; economics uncertain
NfL	IP-MS	High analytical specificity	Reference comparison and standardization	Reference / research laboratories	Low throughput/technical complexity limit access	Efficiency depends on future scalability
	IMR	Sensitive quantification	Exploratory neurodegeneration studies	Research settings	Limited reproducibility and external validation	Commercial scalability uncertain
GFAP	Label-free biosensing (e.g., LSPR-based optical sensors)	Rapid, miniaturizable detection	Proof-of-concept development	Experimental research	Limited clinical validation and regulatory readiness	Potentially low cost; feasibility unproven
Multiplex biomarkers	NULISA and other multiplex immunoassay/biosensor platforms	Broad analyte coverage	Multimarker discovery and phenotyping	Research settings	Limited translational readiness/cutoff harmonization	Economic value depends on scalability

This table presents analytical platforms for Aβ, p-tau, NfL, and GFAP across different stages of translational development. The two-layer structure distinguishes translationally advanced platforms from research-stage or emerging platforms. Diagnostic performance metrics are reported in Table 3. AD, Alzheimer’s disease; BBMs, blood-based biomarkers; Aβ, amyloid-β; CSF, cerebrospinal fluid; PET, positron emission tomography; ELISA, enzyme-linked immunosorbent assay; ECLIA, electrochemiluminescence immunoassay; ECL, electrochemiluminescence; MSD, Meso Scale Discovery; Simoa, single-molecule array; RUO, research use only; CLEIA, chemiluminescent enzyme immunoassay; HISCL, high-sensitivity chemiluminescence enzyme immunoassay system; LC-MS/MS, liquid chromatography–tandem mass spectrometry; IP-MS, immunoprecipitation–mass spectrometry; eMTBR-tau243, endogenously cleaved microtubule-binding-region-containing residue 243; IMR, immunomagnetic reduction; SMID, single molecule immune detection; LSPR, localized surface plasmon resonance; NULISA, nucleic acid linked immuno-sandwich assay; NfL, neurofilament light chain; GFAP, glial fibrillary acidic protein; p-tau, phosphorylated tau.

Nevertheless, several factors currently limit the clinical adoption of plasma Aβ42/40. Compared with CSF Aβ42/40, the magnitude of plasma reduction is smaller in individuals with established brain amyloid pathology (Verberk et al., 2018). Although many studies report associations between plasma Aβ42/40 and cerebral amyloid deposition, weaker correlations have also been observed across populations, limiting its use as a standalone measure of amyloid burden (Rippon et al., 2022; Cogswell et al., 2025). Therefore, plasma Aβ42/40 is currently best positioned as a preliminary risk-assessment and probability-modifying biomarker in clinically selected individuals, particularly when combined with tau-related biomarkers.

### 2.2 Tau axis: tracking tangle pathology

Among plasma tau biomarkers, p-tau181, p-tau217, and p-tau231 represent the most extensively studied candidates, although their biological associations and clinical applications differ (Lan et al., 2025). Plasma phosphorylated tau markers provide information related to AD-associated pathological processes; however, their interpretation requires consideration of clinical context, assay characteristics, and reference standards (Thijssen et al., 2021; Janelidze et al., 2023; Jack et al., 2024). Among these markers, p-tau181 has accumulated substantial clinical evidence and remains widely evaluated (Thijssen et al., 2021; Janelidze et al., 2023). Plasma p-tau231 appears to capture earlier amyloid-associated changes, although its incremental clinical value requires further validation (Ashton et al., 2022; Montoliu-Gaya et al., 2023). In contrast, p-tau217 demonstrates stronger associations with amyloid and tau pathology across disease stages and has emerged as one of the leading candidates for clinical translation. Nevertheless, its clinical interpretation remains context-dependent and should be integrated with clinical information and complementary biomarkers rather than being considered an independent diagnostic measure (Thijssen et al., 2021; Brum et al., 2023; Jack et al., 2024). Beyond currently established plasma p-tau markers, emerging tau species such as endogenously cleaved microtubule-binding-region-containing residue 243 (eMTBR-tau243) may provide additional information regarding tau tangle pathology. However, unlike plasma p-tau181 and p-tau217, eMTBR-tau243 remains primarily a research biomarker, and its clinical applicability requires validation in larger and diverse cohorts (Horie et al., 2025).

Plasma p-tau assay technologies have advanced in analytical sensitivity, automation, and clinical compatibility (Table 2). Current translational efforts increasingly focus on platforms capable of balancing analytical performance with routine laboratory feasibility, including conventional immunoassays (e.g., ELISA), ultrasensitive immunoassays (e.g., Simoa), automated ECLIA platforms, and emerging MS-based approaches. ELISA-based approaches have historically contributed to plasma tau biomarker research, but their limited sensitivity and analytical variability have restricted clinical translation relative to ultrasensitive and automated platforms (Thijssen et al., 2021; Leuzy et al., 2022). Head-to-head comparisons have demonstrated that plasma p-tau assay performance varies across platforms and assay designs. Comparative studies of multiple p-tau181 and p-tau217 assays indicate that differences in antibody configuration, epitope selection, and calibration strategies contribute to variation in absolute concentrations and cutoff values. Despite these analytical differences, p-tau217 assays have demonstrated consistent diagnostic associations across cohorts, although performance remains dependent on assay design and reference standards (Janelidze et al., 2023; Wojdała et al., 2024). IMR-based assays have been explored for plasma tau measurement (Yang et al., 2017), although evidence for their application to p-tau217 remains limited (Janelidze et al., 2023). MS-based approaches enable measurement of multiple tau species, including emerging biomarkers such as eMTBR-tau243, with high analytical specificity and provide valuable mechanistic insights; however, their current clinical application remains limited by technical complexity, specialized infrastructure, and lower scalability compared with automated immunoassay platforms (Barthélemy et al., 2024). Mid-region-directed p-tau181 assays have been developed to evaluate tau-related pathology more specifically, although their clinical advantages require further confirmation (Tagai et al., 2024). Normalized p-tau217 measures, such as %p-tau217 (the ratio of phosphorylated tau217 to its non-phosphorylated counterpart), have been proposed to reduce biological variability by incorporating related non-phosphorylated tau measurements. However, whether these normalized metrics provide additional clinical value beyond absolute p-tau217 concentrations remains uncertain (Bornhorst et al., 2025), and their broader clinical adoption will require multicenter validation. Emerging multiplex approaches, including nucleic acid linked immuno-sandwich assays and novel single molecule immunodetection platforms, remain primarily investigational and require additional clinical validation (Ashton et al., 2025; Jiao et al., 2025).

### 2.3 Neurodegeneration axis: indexing axonal injury

Neurofilament light chain (NfL) is a widely studied marker of neuroaxonal injury and reflects downstream neuronal damage rather than AD-specific molecular pathology (Van Eldik et al., 2024). Plasma NfL correlates with CSF NfL and provides information related to neurodegenerative burden, disease severity, and longitudinal progression across neurodegenerative disorders (Vrillon et al., 2024). Plasma NfL may also contribute to differential characterization among dementia syndromes; nevertheless, because of its limited disease specificity, its principal clinical value lies in assessing neuroaxonal injury and prognosis rather than identifying AD pathology itself (Illán-Gala et al., 2021). In autosomal dominant AD cohorts, plasma NfL elevations can be detected before expected symptom onset, supporting its potential value for tracking early neurodegenerative changes and disease trajectory (Hofmann et al., 2024). However, cerebrovascular and cardiometabolic comorbidities, as well as other neurodegenerative disorders, may independently increase NfL concentrations and complicate interpretation (Liu et al., 2024; Yu et al., 2024). Consistent with this limited disease specificity, associations between plasma NfL and AD-related Aβ/tau pathology or cognitive decline vary across cohorts, supporting its role as a complementary marker of neurodegeneration rather than an independent AD diagnostic biomarker (Pan et al., 2025).

Plasma NfL quantification is performed using several analytical approaches (Table 2), including Simoa, automated CLEIA platforms, IMR, and MS-based methods. Simoa has been extensively applied in research cohorts because of its high analytical sensitivity and established performance in plasma NfL measurement (Liu et al., 2020; Ashton et al., 2021). Automated CLEIA platforms, including Lumipulse, may offer greater compatibility with routine clinical laboratory workflows (Urbano et al., 2024; Martínez-Dubarbie et al., 2025), while other immunoassay platforms, including CLIA-based methods, have also been evaluated for blood NfL measurement (Zondra Revendova et al., 2025). In contrast, IMR and IP-MS-based approaches remain more commonly used in specialized or research settings (Liu et al., 2020; Coulton et al., 2024). Head-to-head evaluations have demonstrated systematic differences in absolute NfL concentrations across assays, indicating that platform-specific thresholds should not be transferred directly between methods (Baiardi et al., 2025). Accordingly, longitudinal measurements should preferably be performed using the same assay platform and interpreted with assay-specific criteria.

### 2.4 Neuroinflammation axis: capturing glial reactivity

Plasma glial fibrillary acidic protein (GFAP) reflects astrocytic activation and has emerged as a biomarker candidate for capturing glial responses associated with AD-related biological changes (Pelkmans et al., 2024; Roveta et al., 2024). Plasma GFAP levels increase across the AD continuum, including preclinical and symptomatic stages, and may provide complementary information for biological stratification and disease progression assessment (Chatterjee et al., 2023). Although plasma GFAP has demonstrated stronger associations with amyloid-related pathology than CSF GFAP in some cohorts, the biological mechanisms underlying this apparent difference remain incompletely understood (Benedet et al., 2021). Therefore, plasma GFAP should be interpreted as a marker of astrocytic activation within a multimodal biomarker framework rather than as an AD-specific indicator. Plasma GFAP elevations may occur years before clinical symptom onset, supporting its potential role in identifying early glial responses before overt clinical progression (Johansson et al., 2023). This limited disease specificity reflects the fact that astrocytic activation can occur in multiple neurological and systemic conditions, highlighting the need for multimodal interpretation. Although some studies have reported strong diagnostic performance in selected early-stage populations, these findings remain dependent on cohort characteristics, assay platforms, and reference standards (Zou et al., 2024). Changes in plasma GFAP during anti-Aβ or anti-tau therapies suggest potential value as a pharmacodynamic marker, although relationships with clinical outcomes remain to be established (Schauer et al., 2024; Bittner et al., 2025). GFAP has been incorporated as an “I” domain biomarker in the revised 2024 AD diagnostic and research framework (Jack et al., 2024).

Plasma GFAP measurement currently relies mainly on ultrasensitive immunoassay platforms (Table 2). Simoa has been extensively used for analytical validation and research applications, whereas automated or semi-automated platforms, including Lumipulse and Ella, are being evaluated to improve scalability and compatibility with clinical laboratory workflows (Fazeli et al., 2024; Kang et al., 2025a; Valera-Barrero et al., 2026). Emerging biosensing approaches remain largely investigational and require additional analytical and clinical validation before broader implementation (Xiao et al., 2025). As with other plasma biomarkers, GFAP interpretation remains dependent on assay characteristics, biological variability, and clinical context.

### 2.5 Sources of uncertainty and real-world variability in BBMs for AD

Blood-based biomarkers for AD are subject to multiple sources of technical variability that arise prior to clinical interpretation and directly influence measurement reliability (Hansson, 2021; Leuzy et al., 2022; Schöll et al., 2025).

Pre-analytical factors, including collection tube type, centrifugation protocols, processing delays, and storage conditions, can substantially affect measured concentrations of plasma Aβ, p-tau, and neurodegeneration markers (Toledo et al., 2011; Schindler et al., 2019; Janelidze et al., 2021; Verberk et al., 2022). Among these, tube type and sample handling contribute measurable variability across assays, with Aβ species being particularly sensitive to pre-analytical conditions, whereas p-tau217 demonstrates relatively greater stability across processing workflows (Bali et al., 2024; Verberk et al., 2025).

At the analytical level, substantial inter-platform variability exists across Simoa, ECLIA, IMR, and MS-based methods, resulting in systematic differences in absolute biomarker concentrations and assay-specific cutoff values (Janelidze et al., 2021; Leuzy et al., 2022; Wojdała et al., 2024; Palmqvist et al., 2025a). Head-to-head and interlaboratory evaluations, including round-robin studies, further demonstrate limited commutability of reference materials across platforms, underscoring a fundamental barrier to harmonization despite robust relative discrimination of AD-related pathology (Barthélemy et al., 2024; Verberk et al., 2025). Even within the same platform, antibody design and calibration strategies can introduce additional systematic variability across assays (Wojdała et al., 2024). Inter-laboratory variation and batch effects remain persistent challenges, although emerging normalization strategies may partially mitigate these issues (Baiardi et al., 2025; Kang et al., 2025a).

Collectively, these technical factors indicate that BBMs are not inherently fixed analytical quantities, but rather assay-dependent measurements that require standardization prior to clinical interpretation–a prerequisite that directly propagates into downstream diagnostic decision-making and must therefore be considered alongside biological and demographic modifiers.

### 2.6 Health economic and cost-effectiveness considerations of BBM platforms

Head-to-head cost comparisons across BBM platforms remain limited, and the economic feasibility of BBM implementation depends not only on assay cost but also on platform scalability, laboratory infrastructure, and intended clinical application (Leelahavarong et al., 2026). While MS-based approaches provide high analytical specificity and reference-level performance, their capital requirements, technical complexity, and limited throughput currently restrict their application mainly to specialized laboratories, analytical validation, and research settings rather than routine high-volume clinical testing (Barthélemy et al., 2024; Janelidze et al., 2024). In contrast, automated immunoassay platforms, including automated CLEIA platforms such as Lumipulse, may offer greater feasibility for clinical laboratories because of higher throughput, compatibility with existing workflows, and potentially lower operational costs, making them suitable for hospital-based BBM implementation and symptom-driven triage pathways (Udeh-Momoh et al., 2022). However, the availability and economic advantages of automated platforms vary across healthcare systems, depending on equipment deployment, reagent accessibility, regulatory status, and local laboratory capacity. ELISA-based assays remain relevant in research and resource-limited settings because of their low equipment requirements, relatively low cost, and experimental flexibility. Although manual operation, lower analytical sensitivity, and limited automation restrict routine clinical adoption, ELISA platforms may still provide practical value in heterogeneous healthcare environments (De Meyer et al., 2020; Leuzy et al., 2022). Ultrasensitive digital immunoassays such as Simoa provide excellent analytical sensitivity and remain valuable for biomarker discovery, cohort studies, and longitudinal research. However, their specialized instrumentation and reagent costs may limit routine large-scale deployment compared with automated clinical analyzers (Leuzy et al., 2022). Therefore, higher analytical sensitivity does not necessarily translate into superior cost-effectiveness for all clinical scenarios. Given that Simoa and automated platforms like Lumipulse have demonstrated comparable diagnostic accuracy for plasma p-tau181 and p-tau217 in symptomatic cohorts (Wojdała et al., 2024), platform selection in real-world clinical settings is largely driven by these distinct economic profiles, throughput requirements, and existing infrastructure compatibility rather than by analytical performance differences alone. However, owing to the lack of publicly available, standardized unit prices for individual commercial kits, the present analysis provides only qualitative cross-platform comparisons of equipment and reagent costs rather than quantitative head-to-head cost-effectiveness modeling for specific products.

Modeling studies illustrate context-dependent cost-effectiveness trade-offs. In a Japanese cohort, generalized linear models combining plasma Aβ42/40 and p-tau217 achieved the best balance of efficacy and cost-effectiveness among 14 preliminary BBM-based selection strategies evaluated for protocol-defined trial enrollment or biomarker-confirmation pathways in the modeling study, though no single model was optimal across all scenarios. Cost-effectiveness improved with higher PET-to-BBM cost ratios, and the minimum cost ratio required for improved cost-effectiveness correlated inversely with amyloid positivity prevalence (Sato et al., 2025). In the Japanese healthcare system, plasma p-tau217 and p-tau217/Aβ42 ratio reduced costs by 34%–79% compared with PET and by −5.6% to 74% compared with inpatient CSF Aβ42/40 (Kurihara et al., 2026). In a U.S. modeling study, a blood test with 88% sensitivity and 89% specificity had estimated value-based prices of $290 for triage and $1,150 for a higher-certainty adjunctive strategy–not stand-alone confirmation–in primary care. These strategies reduced PET or CSF use by 47% and 86%, respectively, while the corresponding prices in specialty care were $450 and $1,950 (Mattke et al., 2025). A recent Chinese cohort study showed that a dual-cutoff plasma p-tau217 strategy could avoid immediate amyloid PET in approximately 84% of memory-clinic patients while reserving PET for indeterminate or high-stakes cases, thereby reducing diagnostic burden and healthcare resource utilization (Wang Y. et al., 2025). Domestic automated CLEIA platforms may have potential advantages for BBM implementation through lower operational costs and broader accessibility within tiered healthcare systems (Liu et al., 2025); however, formal cost-utility analyses incorporating long-term outcomes and reimbursement considerations remain limited in Chinese populations. Cost-effectiveness is not universal across all settings. In Thailand, CSF testing proved cost-effective, whereas BBMs did not, implying that substantial cost reductions would be required for blood-based strategies to achieve economic viability comparable to that of CSF testing (Leelahavarong et al., 2026). Beyond direct costs, BBMs may offer potential sustainability advantages. A preliminary analysis has suggested substantially lower environmental impacts of blood-based approaches compared with CSF collection and amyloid PET, although these estimates require further validation across different healthcare settings (Daly, 2025).

In summary, the economic value of BBMs varies substantially across healthcare systems, driven by local costs, disease prevalence, assay pricing, and clinical use case. Context-specific cost-effectiveness modeling is necessary to guide platform selection and testing strategies.

## 3 Clinical applications of BBMs in AD

**FIGURE 1:**
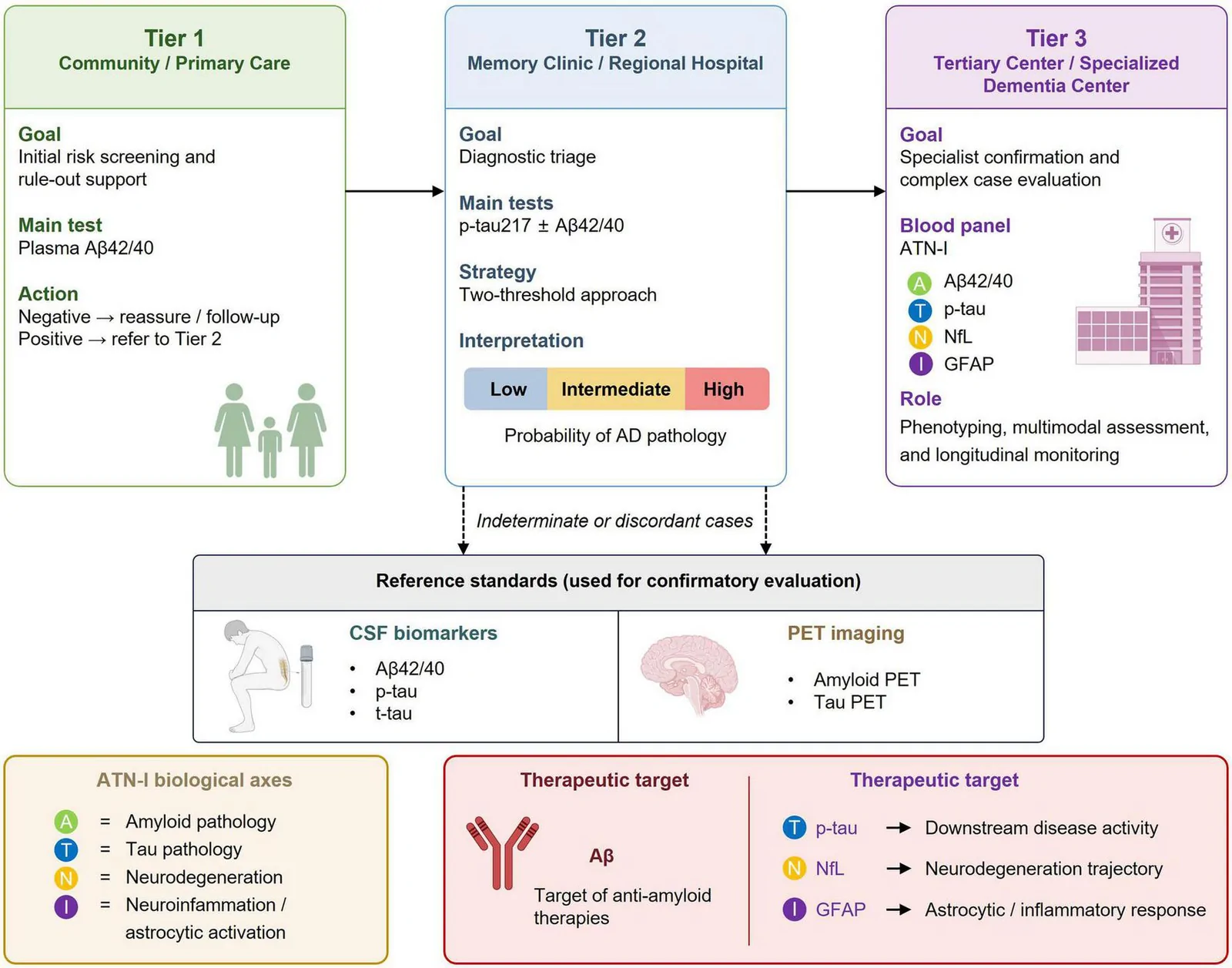
Integrated ATN-I framework for clinical interpretation and tiered application of BBMs in AD. The Figure presents a conceptual, probability-based pathway linking ATN-I biological signals with clinical decision-making across tiered care settings. It is informed by established ATN/ATN-I models, biomarker workflows, and clinical implementation recommendations (Jack et al., 2018; Brum et al., 2023; Imbimbo et al., 2023; Mielke et al., 2024b; Palmqvist et al., 2025a). Tier 1 applies only to individuals with cognitive concerns after an initial clinical assessment and does not refer to testing of unselected populations. BBMs are interpreted as context-dependent probability modifiers within multimodal diagnostic pathways rather than as stand-alone diagnostic tests. The framework is not intended as a standardized diagnostic algorithm or official clinical guideline. Analytical platforms and study-level performance characteristics are presented in Tables 2, 3, respectively. Vector icons were sourced from Biorender. Bin, X. (2026) http://BioRender.com/dvh03ly. BBMs, blood-based biomarkers; AD, Alzheimer’s disease; Aβ, amyloid-β; CSF, cerebrospinal fluid; PET, positron emission tomography; A, amyloid; T, tau; N, neurodegeneration; I, inflammation; p-tau, phosphorylated tau; NfL, neurofilament light chain; GFAP, glial fibrillary acidic protein.

The clinical use of BBMs in AD should be interpreted within a probabilistic and context-sensitive framework (Figure 1). In symptomatic or otherwise clinically selected individuals, BBMs modify the likelihood of AD-related pathology and inform subsequent testing and monitoring rather than independently establishing or excluding a diagnosis (Table 3). Throughout this review, routine clinical use refers to symptom-driven triage or risk assessment in clinically selected individuals after an initial clinical assessment, whereas clinical trial applications refer to protocol-defined eligibility assessment or biomarker-based enrichment.

**TABLE 3:** Clinical performance characteristics of BBMs for AD.

Author (year)	Region	Platform	Cohort	Biomarker	Population	Sample size (n)	Reference standard	Sensitivity (95% CI)	Specificity (95% CI)	AUC (95% CI)	Reported cutoff	Study-level interpretation
A. Amyloid-β (Aβ42/40)												
De Meyer et al., 2020	Europe	EUROIMMUN ELISA	–	Plasma Aβ42/40	Nondemented cohort (CU and aMCI)	199	Amyloid PET	78% (62%–90%)	75% (68%–82%)	0.78 (0.72–0.84)	<0.159 (unitless ratio)	Moderate preclinical performance
De Meyer et al., 2020	Europe	Simoa (Amyblood assay)	–	Plasma Aβ42/40	Nondemented cohort (CU and aMCI)	199	Amyloid PET	74% (57%–87%)	80% (72%–86%)	0.79 (0.73–0.85)	<0.230 (unitless ratio)	Performance comparable to ELISA, not clearly superior
Pascual-Lucas et al., 2023	Spain	ABtest-MS	FACEHBI	Plasma Aβ42/40	Individuals with SCD	200	Amyloid PET	86.1% (NR)	80.5% (NR)	0.87 (0.80–0.93)	0.241 (unitless ratio)	Early enrichment utility
Keshavan et al., 2021	UK	LC-MS/MS	Insight 46	Plasma Aβ42/40	Dementia-free adults	441	Amyloid PET	86.6% (NR)	71.9% (NR)	0.817 (0.770–0.864)	0.095 (unitless ratio; assay-specific)	High diagnostic discrimination
Keshavan et al., 2021	UK	Simoa HD-1 (Quanterix)	Insight 46	Plasma Aβ42/40	Dementia-free adults	441	Amyloid PET	45.1% (NR)	78.0% (NR)	0.620 (0.548–0.691)	0.058 (unitless ratio; assay-specific)	Limited discrimination compared with LC-MS/MS
Bun et al., 2023	Japan	CLEIA (HISCL-5000, Sysmex)	–	Plasma Aβ42/40	Memory-clinic patients	174	Amyloid PET	93.9% (NR)	88.1% (NR)	0.949 (NR)	0.0942 (unitless ratio)	High triage performance
Janelidze et al., 2024	Europe	LC-MS/MS	BioFINDER-2	Plasma Aβ42/40	CU	492	CSF Aβ42/40 status	NR	NR	0.849 (0.810–0.887)	NR	Early Aβ accumulation prediction
Janelidze et al., 2024	US	LC-MS/MS	Knight ADRC	Plasma Aβ42/40	CU	283	CSF Aβ42/40 status	NR	NR	0.855 (0.810–0.900)	NR	Robust replication
Wang S. et al., 2025	China	Simoa HD-X (Quanterix)	Cohort I	Plasma Aβ42/40	AD vs. CU	90 vs. 151	Clinical diagnosis with Aβ-PET support	71.70% (NR)	86.10% (NR)	0.793 (NR)	0.030 (unitless ratio)	Cohort-dependent performance
Wang S. et al., 2025	China	Simoa HD-X (Quanterix)	Cohort II	Plasma Aβ42/40	AD vs. CU	126 vs. 123	CSF-supported diagnosis	82.50% (NR)	56.10% (NR)	0.768 (NR)	0.072 (unitless ratio)	Cohort-dependent performance
B. P-tau (p-tau217, %p-tau217, p-tau181, p-tau231)												
Wang S. et al., 2025	China	Simoa HD-X (Quanterix)	Cohort I	Plasma p-tau217	AD vs. CU	90 vs. 151	Clinical diagnosis with Aβ-PET support	95.00% (NR)	96.00% (NR)	0.990 (NR)	0.551 pg/mL	Cohort-dependent performance
Sarto et al., 2025	Spain	Lumipulse G (Fujirebio; automated CLEIA)	–	Plasma p-tau217	Memory-clinic patients	468	CSF-defined Aβ status	89% (NR)	89% (NR)	0.95 (0.93–0.97)	0.253 pg/mL	Clinical diagnostic support
Thijssen et al., 2021	North America	ECL immunoassay (MSD; Lilly)	UCSF/ARTFL	Plasma p-tau217	AD-spectrum vs. normal controls	75 vs. 118	Clinical diagnosis (AD-spectrum vs. normal controls)	95% (NR)	95% (NR)	0.98 (0.95–1.00)	0.31 pg/mL	High diagnostic discrimination
Jiao et al., 2025	China	SMID (AST-Sc-Lite)	–	Plasma p-tau217	AD/aMCI vs. HC	245 vs. 184	Clinical diagnosis (NIA-AA)	NR	NR	0.95 (NR)	4.2 pg/mL	Strong discrimination in Chinese cohort
Pandey et al., 2025	Peru	Simoa HD-X (Quanterix)	GAPP	Plasma p-tau217	AD vs. HC	113 vs. 234	Consensus clinical diagnosis	83.2% (NR; adjusted-model)	70.5% (NR; adjusted-model)	0.828 (0.77–0.87; adjusted-model)	NR	Adjusted-model discrimination
Zhong et al., 2025	China	Lumipulse G (Fujirebio; automated CLEIA)	SCABI-1	Plasma p-tau217	CU + MCI + dementia	182	Amyloid PET	86% (NR)	94% (NR)	0.95 (0.91–0.98)	0.242 pg/mL	High diagnostic discrimination
Zhong et al., 2025	China	Lumipulse G (Fujirebio; automated CLEIA)	SCABI-2	Plasma p-tau217	CU + MCI + dementia	78	Amyloid PET	87% (NR)	94% (NR)	0.96 (0.91–1.00)	0.242 pg/mL	High diagnostic discrimination
Zhong et al., 2025	China	Lumipulse G (Fujirebio; automated CLEIA)	RCP	Plasma p-tau217	CU + MCI + dementia	100	CSF biomarkers	96% (NR)	65% (NR)	0.94 (0.89–0.99)	0.242 pg/mL	High diagnostic discrimination
Janelidze et al., 2024	Europe	LC-MS/MS	BioFINDER-2	Plasma %p-tau217	CU	492	CSF Aβ42/40 status	NR	NR	0.924 (0.894–0.954)	NR	Early Aβ accumulation prediction
Janelidze et al., 2024	US	LC-MS/MS	Knight ADRC	Plasma %p-tau217	CU	283	CSF Aβ42/40 status	NR	NR	0.895 (0.845–0.946)	NR	Robust replication
Wang S. et al., 2025	China	Simoa HD-X (Quanterix)	Cohort II	Plasma p-tau181	AD vs. CU	126 vs. 123	CSF-supported diagnosis	74.60% (NR)	91.90% (NR)	0.894 (NR)	3.054 pg/mL	Cohort-dependent performance
Thijssen et al., 2021	North America	ECL immunoassay (MSD; Lilly)	UCSF/ARTFL	Plasma p-tau181	AD-spectrum vs. normal controls	75 vs. 118	Clinical diagnosis (AD-spectrum vs. normal controls)	93% (NR)	93% (NR)	0.97 (0.94–0.99)	1.45 pg/mL	High diagnostic discrimination across AD-related contrasts
Keshavan et al., 2021	UK	Simoa HD-1 (Quanterix)	Insight 46	Plasma p-tau181	Dementia-free adults	441	Amyloid PET	70.7% (NR)	68.3% (NR)	0.707 (0.646–0.768)	10.8 pg/mL (assay-specific)	Moderate discrimination
Sarto et al., 2025	Spain	Simoa (Quanterix)	–	Plasma p-tau181	Memory-clinic patients	346	CSF-defined Aβ status	85% (NR)	85% (NR)	0.90 (0.87–0.93)	NR	Clinical diagnostic support
Jiao et al., 2025	China	SMID (AST-Sc-Lite)	–	Plasma p-tau181	AD/aMCI vs. HC	245 vs. 184	Clinical diagnosis (NIA-AA)	NR	NR	0.82 (NR)	8.7 pg/mL	Moderate discrimination
Jiao et al., 2025	China	SMID (AST-Sc-Lite)	–	Plasma p-tau231	AD/aMCI vs. HC	245 vs. 184	Clinical diagnosis (NIA-AA)	NR	NR	0.93 (NR)	10.6 pg/mL	Strong discrimination in Chinese cohort
C. Neurodegeneration (NfL)												
Fang et al., 2024	China	Serum direct CLIA (Maccura)	–	Serum NfL	HC vs. AD	85 vs. 52	Clinical diagnosis (NIA-AA)	59.6% (NR)	76.2% (NR)	0.708 (0.619–0.798)	40.9 pg/mL	Modest AD discrimination; non-specific injury marker
Fang et al., 2024	China	Serum direct CLIA (Maccura)	–	Serum NfL	HC vs. MCI	85 vs. 48	Clinical diagnosis	40.0% (NR)	76.2% (NR)	0.577 (0.470–0.684)	40.85 pg/mL	Low, non-significant MCI discrimination
Illán-Gala et al., 2021	US	Simoa HD-1 (Quanterix)	–	Plasma NfL	FTLD vs. AD	167 vs. 43	Clinical diagnosis	NR	NR	0.75 (0.68–0.82)	NR	Moderate FTLD–AD discrimination
Illán-Gala et al., 2021	US	Simoa HD-1 (Quanterix)	–	Plasma NfL	AD vs. HC	43 vs. 55	Clinical diagnosis	NR	NR	0.94 (0.89–0.98)	NR	High AD–HC discrimination
Illán-Gala et al., 2021	US	Simoa HD-1 (Quanterix)	–	Plasma NfL	FTLD vs. HC	167 vs. 55	Clinical diagnosis	NR	NR	0.97 (0.95–0.99)	NR	High FTLD–HC discrimination
Jiao et al., 2025	China	SMID (AST-Sc-Lite)	-	Plasma NfL	AD/aMCI vs. HC	245 vs. 184	Clinical diagnosis (NIA-AA)	NR	NR	0.62 (NR)	104.2 pg/mL	Low aMCI/AD discrimination
D. Neuroinflammation (GFAP)												
Fang et al., 2024	China	Serum direct CLIA (Maccura)	–	Serum GFAP	HC vs. AD	85 vs. 52	Clinical diagnosis (NIA-AA)	90.4% (NR)	82.1% (NR)	0.928 (0.886–0.971)	31.40 pg/mL	High AD discrimination
Fang et al., 2024	China	Serum direct CLIA (Maccura)	–	Serum GFAP	HC vs. MCI	85 vs. 48	Clinical diagnosis	62.2% (NR)	82.1% (NR)	0.706 (0.673–0.847)	32.05 pg/mL	Moderate MCI discrimination
Fang et al., 2024	China	Serum direct CLIA (Maccura)	–	Serum GFAP	MCI vs. AD	48 vs. 52	Clinical diagnosis	71.7% (NR)	71.1% (NR)	0.749 (0.651–0.846)	46.05 pg/mL	Moderate MCI–AD discrimination
Jiao et al., 2025	China	SMID (AST-Sc-Lite)	–	Plasma GFAP	AD/aMCI vs. HC	245 vs. 184	Clinical diagnosis (NIA-AA)	NR	NR	0.79 (NR)	24.4 pg/mL	Moderate discrimination

Performance estimates should not be directly compared across studies because of differences in cohorts, assays, sample matrices, reference standards, and cutoff definitions. Cutoffs are assay- and cohort-specific rather than universal clinical thresholds. Values were extracted as reported, with cohort-specific estimates presented separately and adjusted models retained only when they represented the primary reported model. BBMs, blood-based biomarkers; AD, Alzheimer’s disease; Aβ, amyloid-β; CSF, cerebrospinal fluid; PET, positron emission tomography; ELISA, enzyme-linked immunosorbent assay; Simoa, single-molecule array; SMID, single molecule immune detection; LC-MS/MS, liquid chromatography–tandem mass spectrometry; SCD, subjective cognitive decline; NR, not reported; CU, cognitively unimpaired; HISCL, high-sensitivity chemiluminescence enzyme immunoassay system; MCI, mild cognitive impairment; aMCI, amnesic mild cognitive impairment; HC, healthy controls; ECL, electrochemiluminescence; MSD, Meso Scale Discovery; CLEIA, chemiluminescent enzyme immunoassay; CLIA, chemiluminescence immunoassay; FTLD, frontotemporal lobar degeneration; AUC, area under the receiver operating characteristic curve; CI, confidence interval; p-tau, phosphorylated tau; %p-tau217, biologically normalized ratio derived from phosphorylated tau217 and its non-phosphorylated counterpart; NIA-AA, National Institute on Aging–Alzheimer’s Association; NfL, neurofilament light chain; GFAP, glial fibrillary acidic protein.

### 3.1 Symptom-driven triage and early evaluation in patients with suspected AD

Blood-based biomarkers are increasingly relevant to the clinical evaluation of AD, with their best-supported application currently centered on symptom-driven triage and biomarker-informed evaluation rather than unrestricted population-wide testing. Their clinical use is most defensible in individuals with cognitive symptoms or concerns who have undergone an initial clinical assessment, or in other clearly defined settings with an appropriate pretest probability.

In this context, pretest probability is not merely a background characteristic but a central determinant of diagnostic interpretation. The same biomarker profile may yield substantially different post-test probabilities depending on disease stage, clinical setting, and assay-related variability (Therriault et al., 2024b; Pahlke et al., 2025). For example, a positive plasma p-tau signal in a memory clinic population with high baseline AD likelihood carries a markedly different interpretive weight compared with the same result in a primary care population with low pretest probability (Therriault et al., 2024b; Bouteloup et al., 2025). Accordingly, current clinical guidelines emphasize that BBMs function as context-sensitive probability modifiers rather than standalone diagnostic determinants. The Alzheimer’s Association practice guideline, for instance, proposes that assays with ≥90% sensitivity and ≥75% specificity may support triage in specialized settings, while those meeting both ≥90% thresholds may reduce–but not replace–CSF or PET confirmation in selected cases (Jack et al., 2024; Palmqvist et al., 2025b). Real-world studies have further supported the clinical utility of BBMs in symptomatic populations. Plasma p-tau217-based strategies have demonstrated improved diagnostic performance in memory-clinic cohorts and have shown potential to facilitate patient stratification and referral decisions across primary and secondary care settings (Palmqvist et al., 2024, 2025a). These findings support the integration of BBMs into symptom-driven triage pathways rather than testing of unselected populations.

Among individual plasma biomarkers, p-tau217 has shown strong and consistently reproducible performance for estimating AD-related pathological probability across clinically validated cohorts, with particular value for identifying AD-related pathology and lowering its estimated probability in appropriately selected patients with low pretest probability (Therriault et al., 2024a; Kang et al., 2025b; Palmqvist et al., 2025a; Pandey et al., 2025). In such a panel-based approach, Aβ42/40 provides early information on amyloid accumulation, GFAP reflects astrocytic activation associated with neuroinflammatory processes, and NfL captures downstream neuroaxonal injury, thereby enabling a more comprehensive characterization of AD-related biological changes across heterogeneous patient populations (Bun et al., 2023; Chatterjee et al., 2023; Fang et al., 2024; Janelidze et al., 2024). A clinically sensible strategy is therefore not a single-marker approach, but an ATN-I blood-based panel in which p-tau217 functions as a core marker and Aβ42/40, GFAP, and NfL refine probability assessment and downstream decision-making (Imbimbo et al., 2023).

Importantly, BBMs should be conceptualized as dynamic probability-modifying instruments rather than fixed diagnostic indicators. This probabilistic behavior underpins the emerging two-threshold strategy. In this approach, lower cutoffs prioritize sensitivity to identify a lower-likelihood group, whereas higher cutoffs maximize specificity to identify a higher-likelihood group. Intermediate results define an indeterminate zone requiring further evaluation. Recent studies suggest that such strategies may improve overall diagnostic accuracy to over 90% while reducing indeterminate classifications to approximately 12%–17% of cases (Giacomucci et al., 2025; Lan et al., 2025).

Taken together, this framework positions BBMs as early-stage decision-support tools that enhance clinical stratification, refine diagnostic probability, and guide downstream testing (Table 1), while maintaining their role within a broader multimodal diagnostic workflow that integrates clinical assessment and, when clinically indicated, additional reference-standard evaluation.

### 3.2 Diagnostic support and clarification in AD

As assay performance improves, BBMs are increasingly incorporated into the diagnostic workup of symptomatic patients with suspected AD (Table 3). The strongest current evidence supports their use as adjunctive tools for diagnostic clarification in specialized settings, particularly when interpreted alongside clinical history, cognitive testing, structural imaging, and assessment of alternative causes. In this context, high-performing plasma p-tau217 assays and ratio-based approaches can estimate the likelihood of AD-related pathology, showing high concordance with CSF-based measures in selected cohorts (Barthélemy et al., 2024; Palmqvist et al., 2025a). Accordingly, these tests are more appropriately viewed as tools that may reduce reliance on CSF or PET in selected patients, rather than as full replacements.

Beyond analytical performance and clinical validation, implementation is also shaped by regulatory and real-world use constraints. The first blood test cleared by the U.S. Food and Drug Administration for AD-related pathology was approved for adults aged 55 years or older with signs or symptoms of cognitive decline, with intended use as an adjunct to clinical assessment rather than as a stand-alone diagnostic tool. It is not approved for use in asymptomatic or otherwise unselected populations (Hu et al., 2025; Wisch and Ances, 2025). Within this regulatory and clinical framework, high-precision blood testing may therefore provide biological support for AD diagnosis in selected symptomatic patients, particularly when amyloid- and tau-related abnormalities are concordant, while requiring integration with the broader clinical context (Jack et al., 2024; Hu et al., 2025).

However, this interpretive flexibility also introduces clinically important limitations. BBM-based diagnostic interpretation requires particular caution in several scenarios: (1) borderline or indeterminate BBM results near assay-specific cutoffs; (2) discordant biomarker patterns; (3) atypical or mixed-pathology cases; (4) low-pretest-probability settings; and (5) situations requiring maximal pathological certainty for treatment or trial eligibility (Mattsson-Carlgren et al., 2024; Palmqvist et al., 2025b). In such contexts, CSF or PET-based reference-standard assessment remains preferable.

In clinical practice, BBM results should be integrated with clinical presentation, cognitive assessment, and complementary biomarkers to support diagnostic clarification. Positive results may increase the likelihood of AD-related pathology (Brum et al., 2023, 2024), whereas negative or intermediate results require interpretation according to pretest probability and clinical context (Du et al., 2024; Therriault et al., 2024b). Therefore, CSF or PET-based reference-standard assessments remain important in cases with diagnostic uncertainty, atypical presentations, or when maximal pathological certainty is required (Hazan et al., 2025; Wisch and Ances, 2025). At the individual patient level, this practical decision framework provides actionable guidance.

Beyond A/T biomarkers (Table 1), plasma NfL and GFAP may provide complementary information regarding neurodegeneration and astrocytic activation, respectively, but their interpretation remains dependent on clinical context because neither marker is specific for AD pathology (Benedet et al., 2021; Chatterjee et al., 2023; Imbimbo et al., 2023). Importantly, the 2024 AD diagnostic/research framework should not be reduced to a one-to-one mapping between plasma biomarkers and clinical stages. Rather, ATN-related biomarkers represent biological processes that are related to–but not synonymous with–clinical staging and syndrome classification (Jack et al., 2024).

### 3.3 Treatment monitoring and prognosis

With the recent approval of disease-modifying therapies, longitudinal BBM assessment is increasingly relevant for clinical practice (Table 4). During clinical follow-up, longitudinal BBM changes may provide complementary information regarding biological response, but interpretation should incorporate cognitive trajectories, imaging findings, treatment exposure, and comorbidities (McDade et al., 2022; Sims et al., 2023; Abbas et al., 2025).

**TABLE 4:** Applications of plasma biomarkers in representative clinical trials.

Drug	Registration ID	CADRO category	Plasma biomarker(s)	Role of biomarker	Target population	Trial phase
ALZ-801 (Hey et al., 2024)	NCT04693520	Amyloid beta	p-tau181, Aβ42/40	Primary p-tau181 endpoint; secondary Aβ42/40 endpoint	Early AD / MCI, 50–80 y	Phase II
Lecanemab (Chen S. et al., 2025)	NCT03887455	Amyloid beta	Aβ42/40, p-tau181, GFAP, NfL	Secondary treatment-effect endpoints	Early AD / MCI, 50–90 y	Phase III
Remternetug (Cummings et al., 2025)	NCT05463731	Amyloid beta	p-tau	Aβ-status eligibility criterion	Early AD, 60–85 y	Phase III
Donanemab (Yaari et al., 2025)	NCT05026866	Amyloid beta	p-tau217	p-tau217 enrichment marker and secondary treatment-effect endpoint	Preclinical AD, 55–80 y	Phase III
ACP-204 (Cummings et al., 2025)	NCT06159673	Neurotransmitter receptors	Plasma biomarkers (not specified)	Aβ-status eligibility criterion	AD with psychiatric symptoms, 55–95 y	Phase III
AR1001 (Cummings et al., 2025)	NCT05531526	Synaptic plasticity/neuroprotection	p-tau181	Secondary treatment-effect endpoint	MCI, 55–90 y	Phase III
Simufilam (Cummings et al., 2025)	NCT04994483	Synaptic plasticity/neuroprotection	p-tau181, p-tau217, GFAP, NfL	Baseline p-tau181 for Aβ-status eligibility; GFAP, NfL, and p-tau217 as additional endpoints	Mild-moderate AD, 50–87 y	Phase III
Buntanetap (Cummings et al., 2025)	NCT06709014	Proteostasis/proteinopathies	p-tau217	p-tau217-positive enrollment criterion	Early AD, 55–85 y	Phase III
Metformin (Cummings et al., 2025)	NCT04098666	DTT small molecule	Plasma biomarkers (not specified)	Secondary treatment-effect endpoint	MCI, 55–90 y	Phase III
Nilotinib (Cummings et al., 2025)	NCT05143528	DTT small molecule	Aβ42, Aβ40, t-tau, p-tau	Secondary treatment-effect endpoint	Early AD, 55–85 y	Phase III
Semaglutide (Cummings et al., 2025)	NCT05891496	DTT small molecule	Aβ42/40, p-tau217/np-tau217	Aβ-status eligibility criterion	MCI / mild AD, 55–75 y	Phase III
Tricaprilin (Cummings et al., 2025)	NCT05809908	DTT small molecule	Aβ42/40, p-tau217, GFAP, NfL	Eligibility criterion and additional endpoints	Mild–moderate AD, 55–85 y	Phase III
Valiltramiprosate (Cummings et al., 2025)	NCT06304883	DTT small molecule	Aβ42, Aβ40, p-tau181, GFAP, and NfL	Secondary treatment-effect endpoint	Early AD, 55–85 y	Phase III
Rotigotine + Rivastigmine (Cummings et al., 2025)	NCT06702124	Cognitive enhancer	NfL, Aβ42, p-tau	Additional treatment-monitoring endpoints	Moderate AD, 55–85 y	Phase III

The use of BBMs in trials should not be interpreted as evidence of validated surrogate outcomes for individual-level clinical benefit. CADRO, Common Alzheimer’s Disease Research Ontology; DTT, Disease-targeted therapy; NCT, ClinicalTrials.gov identifier (“NCT number”); AD, Alzheimer’s disease; MCI, mild cognitive impairment; Aβ, amyloid-β; t-tau, total tau; p-tau, phosphorylated tau; np-tau217, non-phosphorylated tau217 (used in normalized ratios such as p-tau217/np-tau217); GFAP, glial fibrillary acidic protein; NfL, neurofilament light chain.

Among currently studied markers, plasma p-tau217 has shown the most consistent longitudinal value across cohorts for tracking progression along the AD continuum and for reflecting treatment-related change in downstream pathology (Ashton et al., 2022). Plasma Aβ42/40 may be especially informative during early cerebral amyloid accumulation, whereas GFAP appears useful for tracking astrocytic reactivity and NfL for broader neuroaxonal injury and prognosis (Ashton et al., 2022; Mattsson-Carlgren et al., 2023; Janelidze et al., 2024; Pan et al., 2025). Within this interpretive constraint, longitudinal patterns should be considered in a marker-specific manner. Emerging tau species such as eMTBR-tau243 may further refine monitoring of tau-tangle biology, but these markers remain investigational (Horie et al., 2025). For practical longitudinal use, interpretation of serial BBM measurements should ideally rely on the same assay platform (Table 3), comparable preanalytical handling, and repeated measurement within a clinically interpretable framework. No universally accepted cross-platform thresholds for longitudinal change currently exist, and apparent biomarker drift may reflect assay differences or intercurrent systemic illness rather than true disease progression (Wojdała et al., 2024; Baiardi et al., 2025). Taken together, these methodological and interpretive considerations highlight that BBM trajectories are most informative when embedded in multimodal assessment frameworks (Table 1).

Overall, the most defensible current conclusion is that BBMs are transitioning from cross-sectional research tools toward clinically useful instruments for triage, adjunctive diagnostic support in selected patients, and longitudinal biological monitoring, but their optimal use remains context-dependent rather than universal. Their value is greatest when embedded in structured care pathways and interpreted in light of pretest probability, confounding factors, and explicit clinical decision points (Jack et al., 2024; Palmqvist et al., 2025b).

### 3.4 Effects of clinical heterogeneity on BBM profiles in AD

Emerging evidence indicates that the diagnostic and prognostic performance of BBMs for AD varies substantially across distinct populations (Table 3), modulated by ancestry, demographic features, and comorbidity burden (Brickman et al., 2021, 2022; Tian et al., 2024; Griswold et al., 2025; Zhong et al., 2025). Among demographic factors, age acts as a powerful effect modifier. Even among cognitively unimpaired individuals, aging is associated with increased plasma NfL and GFAP levels and modest changes in p-tau and Aβ42 concentrations (Bornhorst et al., 2022, 2025; Rodero-Romero et al., 2024; Bouteloup et al., 2025; Pan et al., 2025; Rousset et al., 2026). Such age-related fluctuations blur the boundary between physiological variations and pathological alterations, especially in elderly cohorts. This highlights an urgent demand for age-stratified reference intervals and longitudinal trajectory-based biomarker interpretation (Jack et al., 2024).

Multi-ethnic cohort studies have shown that, although the directionality of biomarker alterations–such as elevated plasma p-tau and reduced Aβ42/40 ratios in AD–remains broadly consistent across racial and ethnic groups, the optimal diagnostic thresholds and effect sizes may differ substantially between populations (Brickman et al., 2025; Griswold et al., 2025; Jin et al., 2026). These differences are thought to result from a complex interplay of genetic background, environmental exposures, and healthcare-system-related factors, rather than reflecting fundamental differences in underlying disease biology (Brickman et al., 2025; Kpelle et al., 2026). This observation underscores the necessity of population-specific calibration, as opposed to the application of universal cut-off values.

Furthermore, common comorbid conditions exert a marked influence on biomarker concentrations and their clinical interpretation. Chronic kidney disease may elevate circulating NfL levels via impaired renal clearance (Berry et al., 2022; Axelsson et al., 2025), while cerebrovascular pathology and mixed neurodegenerative conditions may amplify neuroaxonal injury signals independently of AD-specific processes (Graff-Radford et al., 2022; Twait et al., 2024). Metabolic disorders, including type 2 diabetes and obesity, have also been associated with altered amyloid and tau biomarker profiles, potentially through systemic inflammatory mechanisms (Bermudez et al., 2025; Mielke et al., 2025; Yang et al., 2026). Additionally, age-related changes in blood–brain barrier integrity and reactive gliosis may further modulate plasma GFAP levels, adding another layer of complexity to interpretation in older populations (Bouhrara et al., 2024; Edwards et al., 2025; Liu et al., 2026). Notably, apolipoprotein E (APOE) genotype constitutes a major genetic driver of inter-population biomarker variability. The APOE ε4 allele, the strongest genetic risk factor for late-onset AD, increases plasma amyloid and p-tau concentrations decades before clinical symptom onset (Farrer et al., 1997; Martínez-Dubarbie et al., 2024; Woo et al., 2025), while non-ε4 carriers display weaker biomarker signals even with confirmed AD pathology (Woo et al., 2025). Ancestry-specific differences in APOE allele frequencies further distort universal diagnostic thresholds, indicating that joint adjustment for APOE status alongside age and ancestry is essential to minimize interpretive bias.

Collectively, these findings advocate for a stratified interpretation framework in which BBMs are evaluated with due consideration of ancestry, age, comorbidities, and other demographic variables. Future clinical implementation should prioritize the establishment of clinically adjusted reference ranges and rigorously validated subgroup-specific thresholds. Failure to account for population-specific differences may lead to both diagnostic inaccuracy and inefficient resource use, as universal cutoffs could prompt unnecessary confirmatory testing in some groups while missing cases in others. This consideration further supports the development of stratified interpretation frameworks for equitable application of BBMs across diverse healthcare settings.

### 3.5 Translating BBM frameworks into tiered healthcare systems: implications from China

From a translational perspective, BBM test results should be interpreted within a probabilistic decision framework rather than as binary diagnostic outcomes (Mielke et al., 2024b). Tiered healthcare systems provide a practical context for aligning these probability estimates with stepwise clinical decision-making (Mattke et al., 2023). However, successful implementation of BBMs requires consideration of healthcare infrastructure, diagnostic accessibility, and reimbursement policies within specific healthcare systems. China represents an important example because of its rapidly increasing dementia burden, uneven distribution of specialist resources, and recent efforts to establish a more coordinated national dementia prevention and care framework. The national dementia action strategy released in 2025 emphasizes an integrated continuum encompassing prevention, early cognitive assessment and identification, diagnosis, treatment, rehabilitation, and long-term care, thereby providing a policy context for scalable diagnostic approaches such as BBMs (Luo et al., 2025; Zhang et al., 2025). Within this evolving framework, dementia assessment in China is increasingly shifting toward earlier identification, standardized management, and coordination across primary care institutions, secondary hospitals, and tertiary dementia centers (Gan et al., 2024; Luo et al., 2025; Zhang et al., 2025). Nevertheless, advanced diagnostic resources, including amyloid PET, CSF biomarker testing, and multidisciplinary memory clinics, remain concentrated in tertiary hospitals, creating challenges for large-scale implementation of biological assessment (Mattke et al., 2023). Therefore, BBMs may provide practical value by improving patient stratification and optimizing referral efficiency.

Using China as an illustrative setting, the following tiered pathway is proposed as a conceptual implementation framework rather than a validated national algorithm. Within this conceptual schema, at the initial point of care (e.g., primary care), BBMs may assist in evaluating clinically selected individuals whose cognitive symptoms or concerns persist after an initial clinical assessment (Palmqvist et al., 2024; Erickson et al., 2025). Because patient volume is high and the pretest probability of AD is low in these settings, the primary clinical and economic value of BBMs lies in their high negative predictive value. In selected low-probability cases, a lower-likelihood BBM result may support deferral of immediate specialist referral or reference-standard testing, provided that clinical follow-up and reassessment remain available. Within China’s primary healthcare system, this strategy may be particularly relevant because community healthcare centers and county-level hospitals currently have limited access to specialized dementia diagnostic services. A negative or low-risk BBM profile may help reduce unnecessary referrals, whereas abnormal or uncertain results could trigger upward referral to regional hospitals or tertiary centers through existing two-way referral mechanisms (Mattke et al., 2023; Palmqvist et al., 2024). Plasma Aβ42/40 and GFAP primarily contribute to early biological signal identification (Abdelhak et al., 2022; Rabe et al., 2023); however, their use is constrained by limited specificity and inter-individual variability (Dark et al., 2024; Schöll et al., 2025). Therefore, their role at this level is restricted to supporting escalation decisions rather than defining a diagnosis.

At intermediate clinical levels, a probability-stratified interpretation approach–categorizing patients into low, intermediate, or high likelihood of AD-related biological changes–is preferable to rigid thresholds (Brum et al., 2024; Palmqvist et al., 2024). Here, plasma p-tau217 serves as the central marker for AD-related pathology, while Aβ42/40, GFAP, and NfL provide complementary information across the amyloid, neuroinflammatory, and neurodegenerative axes to guide the need for further evaluation (Pais et al., 2023; Figdore et al., 2024; Mielke et al., 2024b; Schöll et al., 2025). For regional hospitals and memory clinics in China, BBMs may serve as intermediate decision-support tools by prioritizing patients who require confirmatory PET or CSF testing and improving consistency of diagnostic assessment across institutions (Mattke et al., 2023; Lan et al., 2025). This approach may be particularly valuable given the limited availability and high cost of advanced biomarker examinations in many regions.

At the specialist care level, BBMs could, in principle, support diagnostic clarification and disease characterization, particularly in cases with diagnostic uncertainty or atypical presentations (Mielke et al., 2024b). Nevertheless, confirmation of AD pathology continues to rely on established reference modalities, including CSF biomarkers and PET, when clinically required (Hansson, 2021; Park et al., 2024; Palmqvist et al., 2025b). In tertiary hospitals and specialized dementia centers, where confirmatory diagnostics and disease-modifying treatment pathways are more commonly available, BBMs may additionally facilitate longitudinal biological monitoring and treatment evaluation (Mattsson-Carlgren et al., 2024; Chen S. et al., 2025). This integration is especially relevant as disease-modifying therapies enter clinical practice, for which these centers are best positioned to deliver multimodal workflows.

Importantly, this implementation framework does not introduce a new biomarker classification system but operationalizes the established ATN-I biological framework within tiered clinical pathways (Jack et al., 2018, 2024; Imbimbo et al., 2023). Plasma markers serve as peripheral proxies of A/T/N/I-related processes and should not be equated with formal biological staging or clinical syndrome assignment. This structure preserves the biological universality of ATN-I while enabling context-specific implementation (Figure 1). From a health economic perspective, tiered BBM deployment should align with platform-specific cost-effectiveness: automated CLEIA platforms may be considered in primary-care pathways after cognitive concern has been established and an initial clinical assessment completed, where their lower per-test cost and high throughput may help prioritize specialist referral and subsequent testing. In specialist and tertiary centers, ultrasensitive platforms or MS-based assays may complement triage panels for refined biological characterization, with CSF and PET reserved for reference-standard evaluation in indeterminate, discordant, or clinically high-stakes cases. For China and other healthcare systems with heterogeneous resources, broader implementation further depends on referral capacity, memory clinic coverage, local assay standardization, domestic kit commercialization, and incremental reimbursement coverage for BBM panels.

A major barrier to BBM implementation in China is the absence of a unified reimbursement pathway. Although cognitive assessment, neuroimaging, and conventional laboratory examinations are covered under different regional medical insurance schemes, reimbursement policies for emerging BBM assays have not yet been standardized nationally (Mattke et al., 2023; Roth et al., 2023). The clinical adoption of BBMs will therefore depend not only on diagnostic performance but also on health economic evidence demonstrating their ability to reduce unnecessary PET/CSF procedures, improve referral efficiency, and support earlier intervention. Beyond reimbursement considerations, implementation feasibility also depends on the availability of validated assays, laboratory capacity, and integration with existing diagnostic workflows. The emergence of domestically developed BBM platforms may facilitate clinical translation by improving local accessibility and compatibility with regional laboratory infrastructure. Recent studies using Chinese cohorts have demonstrated promising performance of plasma p-tau217 and related biomarker assays, including chemiluminescence-based immunoassay platforms and single-molecule detection technologies (Jiao et al., 2025; Lan et al., 2025; Liu et al., 2025). However, most plasma AD biomarker assays in China remain at different stages of clinical validation and regulatory translation, and the availability of nationally approved diagnostic products remains limited. Broader adoption of these platforms will therefore require multicenter validation, standardized reporting procedures, quality-control frameworks, and regulatory approval as medical diagnostic devices.

Thus, BBMs should be considered as context-dependent decision-support tools embedded within existing healthcare pathways rather than standalone diagnostic replacements (Mielke et al., 2024b; Schöll et al., 2024; Kang et al., 2025b).

## 4 Clinical trial applications of BBMs in AD

With the increasing development of disease-modifying therapies, BBMs are transitioning from exploratory biomarkers toward practical tools for clinical trial design, including participant selection, biological stratification, pharmacodynamic assessment, and longitudinal monitoring. Unlike routine clinical diagnosis, trial applications require standardized biomarker interpretation and predefined analytical procedures to ensure reliable identification of treatment-relevant biological changes (Mattsson-Carlgren et al., 2024; Cummings et al., 2025).

### 4.1 Longitudinal evidence supporting clinical trial design and biomarker-based enrichment

Although longitudinal cohort studies are not equivalent to randomized clinical trial evidence, they provide essential biological foundations for selecting appropriate biomarkers and defining trial populations. Current evidence indicates that clinical validation and longitudinal utility of BBMs vary across disease stages, assay platforms, and study populations (Table 3), informing their selection for biomarker-based enrichment and monitoring strategies in AD clinical trials (Ashton et al., 2022; Chatterjee et al., 2023; Janelidze et al., 2023; Clemmensen et al., 2025; Pan et al., 2025).

In prevention trials, plasma Aβ42/40 may facilitate enrichment of individuals with early amyloid abnormalities, although its role remains primarily supportive rather than diagnostic (Schindler et al., 2019; Trelle et al., 2025); such protocol-defined enrichment strategies may improve statistical efficiency and reduce the burden of eligibility assessment and confirmatory testing (Verberk et al., 2018; Schindler et al., 2019). In contrast, plasma p-tau217 is increasingly prioritized for trial enrichment because it enables identification of biologically defined AD populations and provides longitudinal information relevant to disease progression (Ashton et al., 2022; Mattsson-Carlgren et al., 2023; Insel et al., 2025; Ossenkoppele et al., 2025). In prodromal and early symptomatic cohorts, repeated p-tau217 measurements may capture heterogeneity in biological progression rates and support adaptive trial designs by facilitating stratification of participants according to disease trajectory (Du et al., 2024; Kirsebom et al., 2025). However, interpretation of longitudinal biomarker changes remains dependent on assay platforms, population characteristics, and validated criteria for defining meaningful within-person change (Wojdała et al., 2024; Baiardi et al., 2025).

Beyond individual biomarkers, multimarker approaches incorporating p-tau217, Aβ42/40, GFAP, and NfL are increasingly considered for improving biological characterization and trial stratification. However, these panels should be regarded as enrichment and monitoring tools rather than direct substitutes for clinical outcomes (Ferreira et al., 2023; Abbas et al., 2025).

### 4.2 Biomarker outcomes in randomized treatment trials

In randomized clinical trials (Table 4), BBMs are increasingly incorporated as secondary, exploratory, or pharmacodynamic endpoints (McDade et al., 2022; Sims et al., 2023; van Dyck et al., 2023). Across anti-amyloid and anti-tau intervention studies, reductions in plasma p-tau species and GFAP, as well as attenuated increases in NfL, have been observed in parallel with amyloid clearance and clinical efficacy signals, suggesting that BBMs capture downstream biological responses to therapy (McDade et al., 2022; Chen S. et al., 2025).

Among currently available BBMs, plasma p-tau217 has demonstrated consistent responsiveness to amyloid-targeting interventions and is increasingly evaluated as a marker of treatment-related biological change (Ashton et al., 2022; Mattsson-Carlgren et al., 2023). Plasma GFAP may provide complementary information regarding astrocytic responses, whereas NfL reflects broader neuroaxonal injury associated with disease progression (Chatterjee et al., 2023; Pan et al., 2025).

However, these biomarker changes should be interpreted primarily as pharmacodynamic or exploratory outcomes rather than validated surrogate endpoints for clinical efficacy (Abbas et al., 2025; Ferreira et al., 2026). Although they indicate target engagement and modulation of disease-related pathways, current evidence does not support a consistent quantitative relationship between biomarker change and individual-level clinical benefit across therapies and disease stages. Recent therapeutic trials further emphasize the distinction between biological response and clinical outcome. For example, reductions in plasma biomarkers following anti-amyloid treatment demonstrate target engagement but cannot yet independently predict cognitive benefit at the individual level (McDade et al., 2022; van Dyck et al., 2023; Abbas et al., 2025).

### 4.3 Clinical trial applications and future implementation of BBMs

Completed trials have established the feasibility of using BBMs for protocol-defined participant eligibility assessment and biomarker-based enrichment in trial recruitment, as well as longitudinal follow-up (Mattsson-Carlgren et al., 2023; Yaari et al., 2025). Moreover, ongoing and planned clinical studies increasingly incorporate BBMs (Table 4), in order to identify individuals with underlying AD pathology and reduce reliance on CSF or PET-based preliminary eligibility assessment (Cummings et al., 2025; Hu et al., 2025; Palmqvist et al., 2025a).

Plasma p-tau217, Aβ42/40, and multimarker panels are being used to improve recruitment efficiency in early intervention trials by identifying biologically defined AD populations (Mattsson-Carlgren et al., 2024; Yaari et al., 2025). In prevention trials, this strategy may be particularly valuable because pathological changes precede clinical symptoms by many years, creating a need for scalable approaches to identify individuals most likely to benefit from intervention (Jia et al., 2024; Ossenkoppele et al., 2025). Longitudinal biomarker monitoring may further enhance future trial designs by enabling repeated assessment of biological responses with minimal invasiveness. However, interpretation of serial measurements requires consistent assay platforms, standardized preanalytical procedures, and validated approaches for defining meaningful biomarker change (Verberk et al., 2022, 2025; Wojdała et al., 2024).

Future clinical trials will likely integrate BBMs with imaging, cognitive outcomes, and digital assessments to establish multidimensional efficacy profiles. Nevertheless, BBMs should currently be considered supportive biological endpoints rather than replacements for clinical outcomes or validated surrogate markers (Ferreira et al., 2023, 2026; Abbas et al., 2025).

## 5 Conclusion and limitations

By enabling scalable, minimally invasive tracking of ATN-I pathways, BBMs are contributing to AD assessment paradigms. Large cohort studies and regulatory advances support the clinical utility of plasma Aβ42/40, phosphorylated tau (especially p-tau217), NfL, and GFAP for probability-based risk refinement, biological characterization, adjunctive diagnostic support in clinically selected individuals, and longitudinal monitoring.

However, several interrelated challenges continue to limit the clinical translation of BBMs. Although emerging modeling studies have provided preliminary evidence regarding their economic value, comprehensive evaluation across diverse healthcare systems remains necessary to determine their long-term clinical and economic impact. Population-level differences driven by ancestry, comorbidities, and demographic factors further limit the generalizability of fixed cutoff strategies and highlight the need for context-dependent interpretation. In addition, biological complexity, including overlapping neurodegenerative and vascular pathologies, complicates the direct attribution of biomarker signals to disease-specific processes. Crucially, while BBMs demonstrate strong concordance with CSF and PET biomarkers in selected cohorts, they are not yet sufficiently standardized to replace these reference modalities in routine clinical practice.

Future implementation will require integration of analytical validation, clinical workflows, and healthcare-system considerations to ensure reliable and equitable use of BBMs. To bridge the gap between biological discovery and clinical application, this review provides a translational framework linking clinical applicability, probabilistic interpretation, and tiered implementation of BBMs.

Ultimately, BBMs should not be conceptualized as standalone diagnostic replacements, but as context-sensitive, probabilistic tools embedded within multimodal diagnostic frameworks. Their optimal clinical value will emerge from integration with clinical assessment, neuroimaging, and fluid biomarkers, supporting a more biologically informed and equitable approach to AD diagnosis and management.
